# Classification of Fruits Using Computer Vision and a Multiclass Support Vector Machine

**DOI:** 10.3390/s120912489

**Published:** 2012-09-13

**Authors:** Yudong Zhang, Lenan Wu

**Affiliations:** School of Information Science and Engineering, Southeast University, Nanjing 210096, China; E-Mail: zhangyudongnuaa@gmail.com

**Keywords:** fruit classification, principal component analysis, color histogram, Unser's texture analysis, mathematical morphology, shape feature, multi-class SVM, kernel SVM, stratified cross validation

## Abstract

Automatic classification of fruits via computer vision is still a complicated task due to the various properties of numerous types of fruits. We propose a novel classification method based on a multi-class kernel support vector machine (kSVM) with the desirable goal of accurate and fast classification of fruits. First, fruit images were acquired by a digital camera, and then the background of each image was removed by a split-and-merge algorithm; Second, the color histogram, texture and shape features of each fruit image were extracted to compose a feature space; Third, principal component analysis (PCA) was used to reduce the dimensions of feature space; Finally, three kinds of multi-class SVMs were constructed, *i.e.*, Winner-Takes-All SVM, Max-Wins-Voting SVM, and Directed Acyclic Graph SVM. Meanwhile, three kinds of kernels were chosen, *i.e.*, linear kernel, Homogeneous Polynomial kernel, and Gaussian Radial Basis kernel; finally, the SVMs were trained using 5-fold stratified cross validation with the reduced feature vectors as input. The experimental results demonstrated that the Max-Wins-Voting SVM with Gaussian Radial Basis kernel achieves the best classification accuracy of 88.2%. For computation time, the Directed Acyclic Graph SVMs performs swiftest.

## 1. Introduction

Recognizing different kinds of vegetables and fruits is a difficult task in supermarkets, since the cashier must point out the categories of a particular fruit to determine its price. The use of barcodes has mostly ended this problem for packaged products but given that most consumers want to pick their products, they cannot be prepackaged, and thus must be weighed. A solution is issuing codes for every fruit, but the memorization is problematic leading to pricing errors. Another solution is to issue the cashier an inventory with pictures and codes, however, flipping over the booklet is time consuming [1].

Some alternatives were proposed to address the problem. VeggieVision was the first supermarket produce recognition system consisting of an integrated scale and image system with a user-friendly interface [2]. Hong *et al.* [3] employed morphological examination to separate walnuts and hazelnuts into three groups. Baltazar *et al.* [4] first applied data fusion to nondestructive image of fresh intact tomatoes, followed by a three-class Bayesian classifier. Pennington *et al.* [5] used a clustering algorithm for classification of fruits and vegetables. Pholpho *et al.* [6] used visible spectroscopy for classification of non-bruised and bruised longan fruits, and combined this with principal component analysis (PCA), Partial Least Square Discriminant Analysis (PLS-DA) and Soft Independent Modeling of Class Analogy (SIMCA) to develop classification models.

The aforementioned techniques may have one or several of the following shortcomings: (1) they need extra sensors such as a gas sensor, invisible light sensor, and weight sensor. (2) The classifier is not suitable to all fruits, *viz.*, it can only recognize the varieties of the same category. (3) The recognition systems are not robust because different fruit images may have similar or identical color and shape features [7].

Support Vector Machines (SVMs) are state-of-the-art classification methods based on machine learning theory [8]. Compared with other methods such as artificial neural networks, decision trees, and Bayesian networks, SVMs have significant advantages because of their high accuracy, elegant mathematical tractability, and direct geometric interpretation. Besides, they do not need a large number of training samples to avoid overfitting [9].

In this paper, we chose an image recognition method which only needs a digital camera. To improve the recognition performance, we proposed a hybrid feature extraction technique which combines the color features, Unser's texture, and shape features, followed by the principal component analysis (PCA) to reduce the number of features. Three different multi-class SVMs were used for multi-class classification. We expect this method will solve the fruit classification problem.

The rest of the paper is organized as follows: Section 2 discusses the methods used in this paper. Section 2.1 shows the split-and-merge algorithm for fruits extraction; Section 2.2 gives the descriptors of fruits with respect to the color component, shape component, and texture component. In addition, PCA was introduced as a methodology to reduce the number of features used by the classifiers; Section 2.3 introduced in the kernel SVM, and then gives three schemes for multi-class SVMs, including Winner-Take-All SVM (WTA-SVM), Max-Wins-Voting (MWV-SVM), and Directed Acyclic Graph SVM (DAG-SVM); Section 3 shows the use of 1,653 images of 18 different types of fruits to test our method; and lastly Section 4 is devoted to conclusions.

## 2. Methods

### 2.1. Image Segmentation with the Split-and-Merge Algorithm

First, we use image segmentation techniques to remove the background area since our research only focuses on the fruits. We choose a split-and-merge algorithm, which is based on a quadtree partition of an image. This method starts at the root of the tree that represents the whole image. If it is found inhomogeneous, then it is split into four son-squares (the splitting process), and so on so forth. Conversely, if four son-squares are homogeneous, they can be merged as several connected components (the merging process). The node in the tree is a segmented node. This process continues recursively until no further splits or merges are possible.

**Figure 1.**
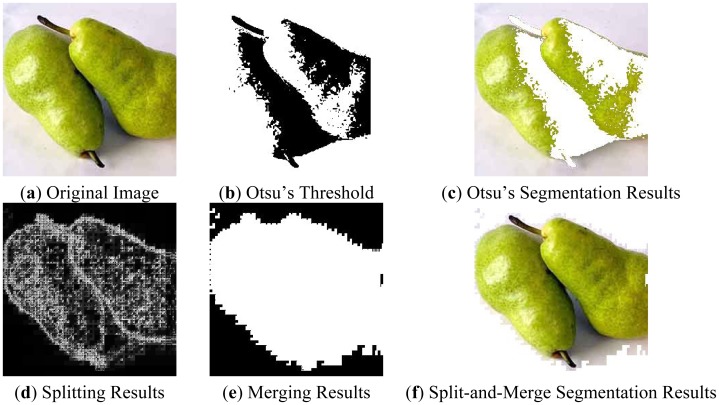
Comparison of Otsu's Method with split-and-merge segmentation.

Figure 1 gives an example. Here the non-uniform light source causes color fluctuations on the surface of thes pear and background, therefore the gray value distributions of both pears and background mix together. Figure 1(b) shows the optimal threshold found by Otsu's method [10]. Figure 1(c) shows the fruits extracted from the Otsu threshold. Apparently the Otsu segmentation only extracts half of the fruits area.

Figure 1(d–f) show our method. The splitting process splits the image to homogeneous small squares (Figure 1(d)) according to the splitting rules, and then combines the connected squares according to the merging rules (Figure 1(e)). The final extraction (Figure 1(f)) shows this split-and-merge process neatly extracts the whole area of fruits.

### 2.2. Feature Extraction and Reduction

We propose a hybrid classification system based on color, texture, and appearance features of fruits. Here, we suppose the fruit images have been extracted by split-and-merge segmentation algorithm [11,12].

#### 2.2.1. Color Histogram

At present, the color histogram is employed to represent the distribution of colors in an image [13]. The color histogram represents the number of pixels that have colors in a fixed list of color range that span the image's color space [14].

For monochromatic images, the set of possible color values is sufficiently small that each of those colors may be placed on a single range; then the histogram is merely the count of pixels that have each possible color. For color images using RGB space, the space is divided into an appropriate number of ranges, often arranged as a regular grid, each containing many similar color values.

**Figure 2.**
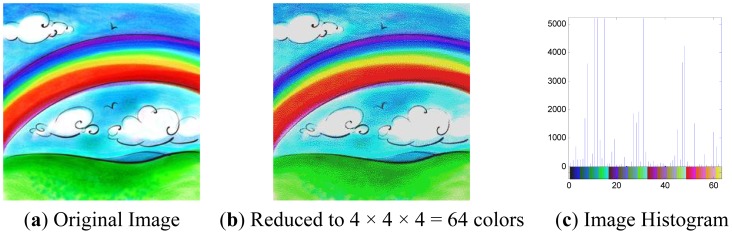
Rainbow image.

Figure 2(a) is an original rainbow image with RGB channels from 0 to 255, so there are totally 256 × 256 × 256 = 2^24^ colors. Figure 2(b) uses four bins to represent each color component, Bins 0, 1, 2, 3 denote intensities 0-63, 64-127, 128-191, 192-255, respectively, so there are in total 4 × 4 × 4 = 64 colors. Figure 2(c) is the histogram of Figure 2(b), where the *x*-axis denotes the index of the 64 colors, and the *y*-axis denotes the number of pixels.

The histogram provides a compact summarization of the distribution of data in an image. The color histogram of an image is relatively invariant with translation and rotation about the viewing axis. By comparing histograms signatures of two images and matching the color content of one image with the other, the color histogram is well suited for the problem of recognizing an object of unknown position and rotation within a scene.

#### 2.2.2. Unser's Texture Features

Gray level co-occurrence matrix and local binary pattern are good texture descriptors, however, they are excessively time consuming. In this paper, we chose the Unser feature vector. Unser proved that the sum and difference of two random variables with same variances are de-correlated and the principal axes of their associated joint probability function are defined. Therefore, we use the sum *s* and difference *d* histograms for texture description [15].

The non-normalized sum and difference associated with a relative displacement (*δ*_1_, *δ*_2_) for an image *I* are defined as:

(1) $$s(k,l;\delta_{1},\delta_{2})=I(k,l)+I(k+\delta_{1},l+\delta_{2})$$

(2) $$d(k,l;\delta_{1},\delta_{2})=I(k,l)+I(k+\delta_{1},l+\delta_{2})$$

The sum and difference histograms over the domain *D* are defined as:

(3) $$h_{s}(i;\delta_{1},\delta_{2})=\text{card}((k,l)\in D,s(k,l;\delta_{1},\delta_{2})=i)$$

(4) $$h_{d}(j;\delta_{1},\delta_{2})=\text{card}((k,l)\in D,d(k,l;\delta_{1},\delta_{2})=j)$$

Next, seven indexes can be defined on the basis of the sum and difference histogram. Those indexes and their corresponding formulas are listed in Table 1. In our method, we firstly abandoned the color information, followed by calculating the 64-bin histogram, and finally obtain the seven indexes.

#### 2.2.3. Shape Features

In this paper we propose eight measures based on mathematical morphology, which are listed in Table 2. The measures can be classified into four groups: (1) The “area”, “perimeter”, and the “Euler number” can be extracted directly from the object; (2) Create a convex hull using Graham Scan method [16] which is the smallest convex polygon that covers the object, then extract the “convex area” and “solidity” features; (3) Create an ellipse that has the same second-moments as the object, then extract the “minor length”, “major length”, and “eccentricity” features. Table 2 illustrates an example of generating the convex hull and the ellipse from original images.

#### 2.2.4. Principal Component Analysis

In total, there are 79 features (64 color features + seven texture features + eight shape features) extracted from a given image. Excessive features increase computation time and storage memory, which sometimes causes the classification process to become more complicated and even decrease the performance of the classifier. A strategy is necessary to reduce the number of features used in classification.

Principal component analysis (PCA) is an efficient tool to reduce the dimensionality of a data set consisting of a large number of interrelated variables while retaining the most significant variations [17]. It is achieved by transforming the data set to a new set of ordered variables according to their degree of variance or importance. This technique has three effects: (1) it orthogonalizes the components of the input vectors so that they are uncorrelated with each other, (2) it orders the resulting orthogonal components so that those with the largest variation come first, and (3) it eliminates the components in the data set that contributes the least variation [18].

**Figure 4.**
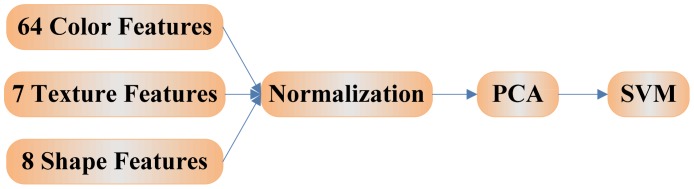
Using normalization before PCA.

It should be noted that the input vectors should be normalized to have zero mean and unity variance before performing PCA, which is shown in Figure 4. The normalization is a standard procedure. Details about PCA are given in [19].

### 2.3. Multiclass Kernel SVMs

#### 2.3.1. Kernel SVM

Traditional linear SVMs cannot separate complicated distributed practical data. In order to generalize it to nonlinear hyperplane, the kernel trick is applied to SVMs [20]. The resulting algorithm is formally similar, except that every dot product is replaced by a nonlinear kernel function. From another point of view, the kernel SVMs allow to fit the maximum-margin hyperplane in a transformed feature space. The transformation may be nonlinear and the transformed space is a higher dimensional space. Though the classifier is a hyperplane in the higher-dimensional feature space, it may be nonlinear in the original input space. Four common kernels [21] are listed in Table 3. For each kernel, there should be at least one adjusting parameter so as to make the kernel flexible and tailor itself to practical data.

SVMs were originally designed for binary classification. Several methods have been proposed for multi-class SVMs, and the dominant approach is to reduce the single multiclass problem into multiple binary classification problems [22]. Three popular types of methods are depicted as follow.

#### 2.3.2. Winner-Takes-All SVM

Assume there are totally *C* (*C* > 2) classes. For the one-versus-all approach, classification of new instances is done by a winner-takes-all (WTA) strategy [23]. We first train *c* different binary SVMs, each one trained to distinguish the data in a single class from the data of all the remaining classes. When applied to a new test data, all the *C* classifiers are run, and the classifier which outputs the largest value is chosen. If there are two identical output values, WTA selects the class with the smallest index.

The mathematical model is described as follow. Given a *p*-dimensional *N*-size training dataset of the form:

(5) $$\{(x_{n},y_{n})\;|\;x_{n}\in R^{p},y_{n}\in \{1,2,\ldots ,C\}\},n=1,2,\ldots ,N$$

where *x_n_* is a *p*-dimensional vector, and *y_n_*_∈_ {1,2,…,*C*} is the class label of each *x_n_*. The classification decision function for *i*th individual binary SVM can be defined as:

(6) $$f_{i}(x)=\sum_{n=1}^{N}y_{n}^{i}\alpha_{n}^{i}k(x_{n},x)-b_{i},i=1,2,\ldots ,C$$

(7) $$y_{n}^{i}=\{\begin{array}{cc} +1 & \text{if}\;x_{n}\in i\text{th class}\\ -1 & \text{otherwise} \end{array}$$

where *N* is the number of training data; *C* is the number of total classes; $y_{n}^{i}\in \{+1,-1\}$ depends on the class label of *x_n_*, if *x_n_* belongs to the *i*th class, $y_{n}^{i}=+1$, otherwise $y_{n}^{i}=-1$; *k*() is the predefined kernel function; $\alpha_{n}^{i}$ is the Lagrange coefficient; and *b_i_* is the bias term. $\alpha_{n}^{i}$ and *b^i^* are obtained by training the *i*th individual SVM. The output of *i*th SVM is the sign function of its decision function, namely:

(8) $$O_{i}(x)=\mathrm{sgn}\;(f_{i}(x))$$

If *f_i_*(*x*) >0, then the output *O_i_*(*x*) is +1, denoting *x* belongs to *i*th class; otherwise output is −1, denoting *x* belongs to other classes.

#### 2.3.3. Max-Wins-Voting SVM

For the one-versus-one approach, classification is done by a max-wins voting (MWV) strategy [23]. First we construct a binary SVM for each pair of classes, so in total we will get *C*(*C*-1)/2 binary SVMs. When applied to a new test data, each SVM gives one vote to the winning class, and the test data is labeled with the class having most labels. If there are two identical votes, MWV selects the class with the smallest index.

The mathematical model is described as follow. The *ij*th (*i* = 1,2, …, *C*-1, *j* = *i* + 1, …, *C*) individual binary SVM is trained with all data in the *i*th class with +1 label and all data of the *j*th class with −1 label, so as to distinguish *i*th class from *j*th class. The decision function of *ij*th SVM is:

(9) $$\begin{array}{l} f_{ij}(x)=\sum_{n=1}^{N_{i}+N_{j}}y_{n}^{ij}\alpha_{n}^{ij}k(x_{n}^{ij},x)-b_{ij}\\ i=1,2,\ldots ,C-1,j=i+1,i+2,\ldots ,C \end{array}$$

(10) $$y_{n}^{ij}=\{\begin{array}{cc} +1 & x_{n}^{ij}\in i\text{th class}\\ -1 & x_{n}^{ij}\in j\text{th class} \end{array}$$

where *N_i_* and *N_j_* denotes the total number of *i*th class and *j*th class, respectively. $y_{n}^{ij}\in \{+1,-1\}$ depends on the class label of $x_{n}^{ij}$. If $x_{n}^{ij}$ belongs to *i*th class, $y_{n}^{ij}=+1$; otherwise $x_{n}^{ij}$ belongs to *j*th class, $y_{n}^{ij}=-1$. $\alpha_{n}^{ij}$ is the Lagrange coefficient; and *b_ij_* is the bias term. $\alpha_{n}^{ij}$ and *b_ij_* are obtained by training the *ij*th individual SVM. The output of *ij*th SVM is the sign function of its decision function, namely:

(11) $$O_{ij}(x)=\mathrm{sgn}(f_{ij}(x))$$

If *f_ij_*(*x*)>0, then the output *O_ij_*(*x*) is +1, denoting *x* belongs to *i*th class; otherwise output is −1, denoting *x* belongs to *j*th class.

#### 2.3.4. Directed Acyclic Graph SVM

A Directed Acyclic Graph (DAG) is a graph whose edges have an orientation and no cycles. The DAG-SVM constructs the individual SVM as the MWV-SVM, however, the output of each individual SVM is explained differently. When *O_ij_*(*x*) is +1, it denotes that *x* does not belong to *j*th class; when *O_ij_*(*x*) is −1, it denotes that *x* does not belong to *i*th class. Therefore, the final decision cannot be reached until the leaf node is reached [24].

**Figure 5.**
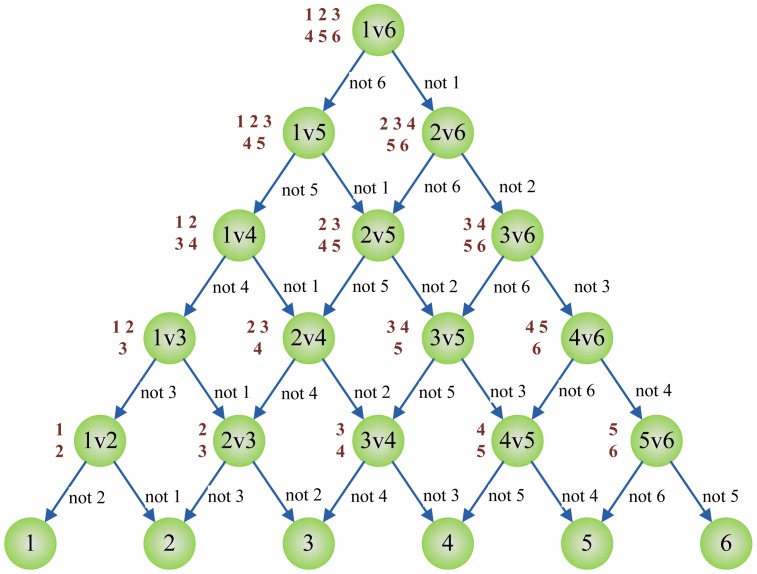
The DAG for finding best class out of six classes.

Figure 5 below shows the DAG for finding the best class out of six given classes. Here, the root node and intermediate nodes represents the individual binary SVM, whereas the leaf nodes represent the output label. Given a test sample *x* starting at the root node, the individual binary SVMs are evaluated. The node is then exited via the evaluation result to either left edge or right edge. The next SVM's function is evaluated again until the leaf node is reached. Therefore, DAG-SVM costs less computation time compared to MWV-SVM. In this case, the MWV-SVM needs to cover all nodes of 15 individual SVMs, yet the DAG-SVM only needs to evaluate only five individual SVMs.

## 3. Experiments & Discussions

The experiments were carried out on a P4 IBM platform with 3 GHz main frequency and 2 GB memory running under the Microsoft Windows XP operating system. The algorithm was developed in-house on the Matlab 2011b (The Mathworks©) platform. The programs can be run or tested on any computer platforms where Matlab is available.

### 3.1. Fruit Recognition System

**Figure 6.**
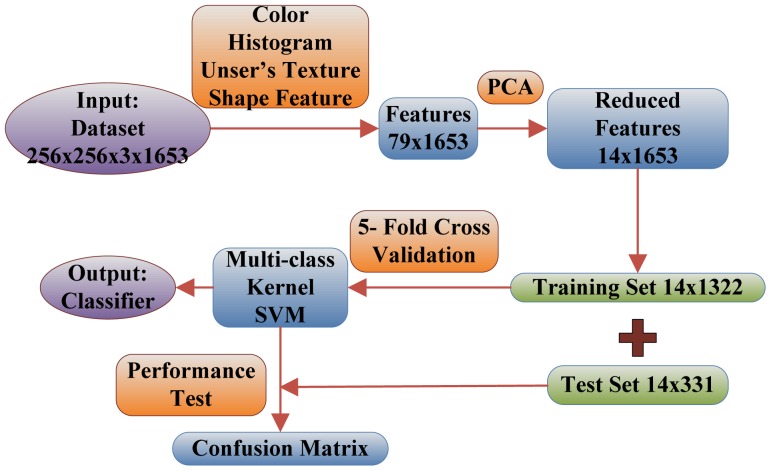
The flowchart of the proposed fruit recognition system.

Below are the steps explaining the flowchart of the proposed fruit recognition system shown in Figure 6.

Numbers in the figure are achieved by experiments below:

**Step 1.** The input is a database of 1,653 images consisting of 18 categories of fruits, and each image size is 256 × 256.
**Step 2.** The 79 features are extracted from each 256 × 256 image. These 79 features contain 64 color features, seven texture features, and eight shape features.
**Step 3.** The 79 features are reduced to only 14 features via PCA, and the selection standard is to preserve 95% energy.
**Step 4.** The 1,653 images are divided into training set (1,322) and test set (331) in the proportion of 4:1. Meanwhile, the training set is treated by 5-fold cross validation.
**Step 5.** The training set is used to train the multi-class SVM. The weights of the SVM are adjusted to make minimal the average error of 5-fold cross validation.
**Step 6.** The test dataset is constructed by randomly sampling in each group, and is used to analyze the performance of the classifier and to calculate the Confusion Matrix. If acceptable, then output the classifier, otherwise return to step 5 to re-train the weights of SVM.

### 3.2. Dataset

The fruit dataset was obtained after six months of on-site data collecting via digital camera and online collecting using images.google.com as the main search engine. The split-and-merge algorithm was used to remove the background areas; later images were cropped to leave the fruit in the center of the image, and finally downsampled to 256 × 256 in size.

The data set comprises 18 different categories: Granny Smith Apples (64), Rome Apples (83), Yellow Bananas (132), Green Plantains (61), Tangerines (112), Hass Avocados (105), Watermelons (72), Cantaloupes (129), Gold Pineapples (89), Passion Fruits (72), Bosc Pears (88), Anjou Pears (140), Green Grapes (74), Red Grapes (45), Black Grapes (122), Blackberries (97), Blueberries (95), and Strawberries (73). In total, there are 1s653 images. Table 4 depicts the samples of different types of fruits in the data set.

### 3.3. Stratified Cross Validation

Typically, a statistical model that deals with the inherent data variability is inferred from the database (*i.e.*, the training set) and employed by statistical learning machines for the automatic construction of classifiers. The model has a set of adjustable parameters that are estimated in the learning phase using a set of examples. Nevertheless, the learning machine must ensure a reliable estimation of the parameters and consequently good generalization, *i.e.*, correct responses to unseen examples, including classifying new images correctly. Hence, the learning device must efficiently find a trade-off between its complexity, which is measured by several variables, such as the effective number of free parameters of the classifier and the feature input space dimension, and the information on the problem given by the training set (e.g., measured by the number of samples).

**Figure 7.**
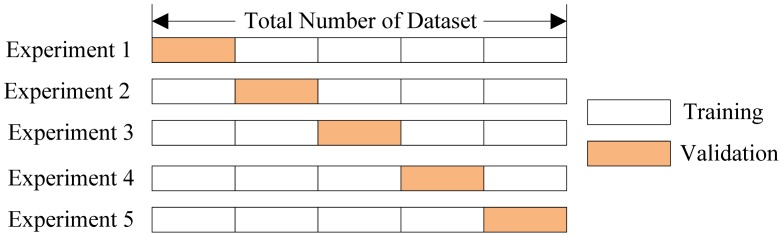
A 5-fold Cross Validation.

Cross validation methods are usually employed to assess the statistical relevance of the classifiers. It consists of four types: Random subsampling, *K*-fold cross validation, leave-one-out validation, and Monte Carlo Cross-Validation [25]. The *K*-fold cross validation is applied due to its simple and easy properties, while using all data for training and validation. The mechanism is to create a *K*-fold partition of the whole dataset, repeat *K* times to use *K*-1 folds for training and a left fold for validation, and finally average the error rates of *K* experiments. The schematic diagram of 5-fold cross validation is shown in Figure 7.

The *K* folds can be purely random partitioned; however, some folds may have quite different distributions from other folds. Therefore, the stratified *K*-fold cross validation was employed, in which every fold has nearly the same class distributions [26]. The folds are selected so that the mean response value is approximately equal in all the folds. In the case of a dichotomous classification, this means that each fold contains roughly the same proportions of the two types of class labels.

Another challenge was to determine the number of folds. If *K* is set too large, the bias of the true error rate estimator will be small, but the variance of the estimator will be large and the computation will be time consuming. Alternatively, if *K* is set too small, the computation time will decrease, the variance of the estimator will be small, but the bias of the estimator will be large [27]. In this study, we empirically determined *K* to be 5 through the trial-and-error method.

### 3.4. PCA Results

**Figure 8.**
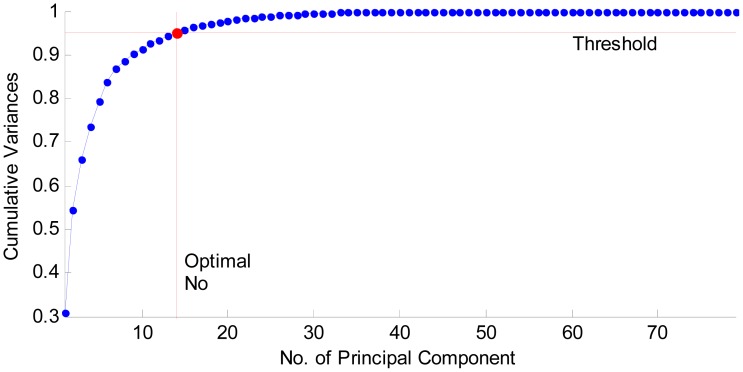
Feature selection via PCA (threshold is set as 95%).

The curve of cumulative sum of variance versus number of reduced vectors via PCA is shown in Figure 8. The detailed data results are listed in Table 5. It shows that merely 14 features can preserve 95.08% energy. The reduced features only cost 17.7% (14/79) of the memory needed for the original 79 features. Consequently, the algorithm can be accelerated remarkably. Seventy nine features are not a burden to the latest computers. But after they are reduced to 14 features, we can accelerate the training and test speed, and meanwhile removing extra features will enhance the classification accuracy.

**Figure 9.**
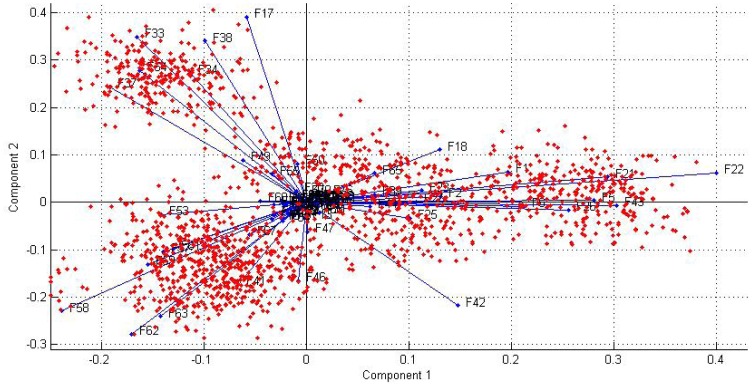
The biplot (red dots represent the samples, and blue lines represent the 79 original features, and x-axis & y-axis represent the two principal components).

The loading plot on principal component 1&2 is shown in Figure 9, from which we can analyze the subsequent role of 79 features for classification. It will guide us to select features more effectively and to identify the relationship between samples and features.

### 3.5. SVM Results

We tested three multi-class SVMs (WTA-SVM, MWV-SVM, and DAG-SVM) using the reduced feature vectors and using 5-fold cross validation. Meanwhile, we chose the Linear (LIN) kernel, *d*th Homogeneous Polynomial (HPOL) kernel, and the Gaussian Radial Basis (GRB) kernel as listed in Table 3. We computed hundreds of simulations in order to estimate the optimal parameters of the kernel functions, such as the order *d* in HPOL kernel and the scaling factor *γ* in GRB kernel.

Tables 6 and 7 show the classification accuracy and calculation time in five-fold cross-validation for those SVMs with optimized parameters, respectively. For LIN kernel, the classification accuracies of MWV-SVM and DAG-SVM are the same of 53.5%, higher than the WTA-SVM of 48.1%; for the HPOL kernel, the classification accuracy of MWV-SVM is 75.6%, higher than WTA-SVM of 61.7% and DAG-SVM of 70.1%; for the GRB kernel, the classification accuracy of MWV-SVM is 88.2%, still higher than WTA-SVM of 55.4% and DAG-SVM of 84.0%. Therefore, the best results are achieved using the GRB kernel MWV-SVM with a classification accuracy of 88.2%.

As for the classification speed, the WTA-SVM is the slowest, for it uses one-*versus*-all strategy so the dataset needed for training is relatively large. The MWV-SVM is faster than WTA-SVM for it uses one-versus-one strategy so every individual binary SVM only takes about 2/18 portion of the data. The DAG-SVM is yet four times faster than MWV-SVM. The reason leans upon that the MWV-SVM needs all 153 individual SVMs to make the final decision, nevertheless the DAG-SVM needs only 17 binary SVMs to complete the equivalent task.

### 3.6. Confusion Matrix

**Figure 10.**
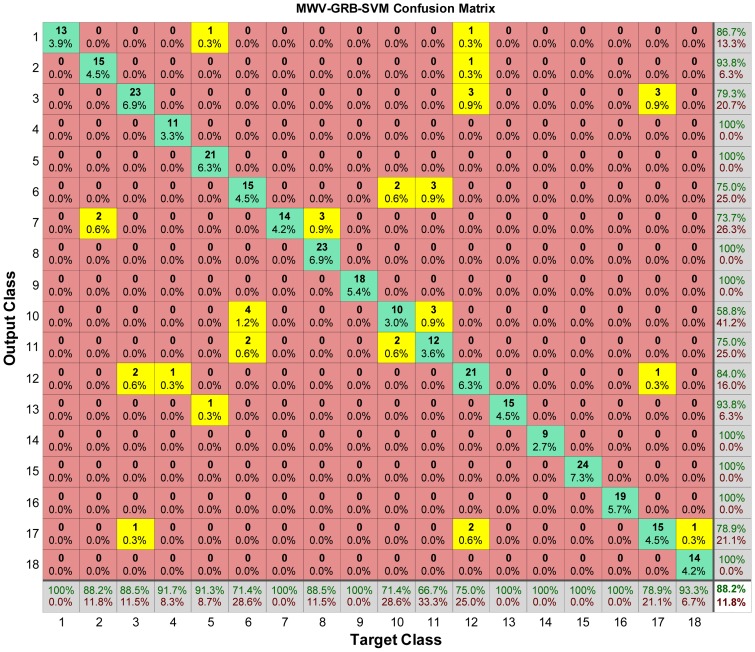
Confusion matrix of GRB kernel MWV-SVM with overall classification accuracy of 88.2%.

The confusion matrix of GRB kernel MWV-SVM is shown in Figure 10. Each column of the matrix represents the instances in target class (actual class), while each row represents the instances in the output class (predicted class). The number in *i*th row and *j*th column represents samples whose target is the *j*th class that was classified as *i*th class. All the misclassification cases are highlighted in yellow.

By looking at the bottom line of Figure 10, we found that 1st class (Granny Smith Apples), 7th class (Water melon), 9th class (Golden Pineapple), 13th class (Green Grape), 14th class (Red Grape), 15th class (Black Grape), 16th class (Black berry) are all recognized successfully.

Notwithstanding, a few types of fruits were not recognized so successfully. The SVM for the 11th class (Bosc Pears) performs the worst. In the test dataset, there are 18 different pictures of Bosc Pears, however, three of them are misclassified as 6th class (Avocado), and another three of them are misclassified as 10th class (Passion Fruits), so the rest are recognized correctly leading to a 66.7% (12/18) success rate. For the 6th class (Avocado), it has twenty-one samples in the test dataset, but four are misclassified as 10th class (Passion Fruits) and two are misclassified as 11th class (Bosc Pears), so with the rest fifteen samples recognized, this give us a 71.43% (15/21) success rate. For the 10th class (Passion Fruits), it has fourteen samples in the test dataset, two are misclassified as 6th class (Avocado), and another two are misclassified as 11th class (Bosc Pears), so with the rest recognized, it give us a 71.43% (10/14) success rate. In other words, the 6th, 10th, and 11th classes are not distinct in the view of SVM, which is a motivation for our future work in order to solve this misclassification.

## 4. Conclusions

This work proposed a novel classification method based on multi-class kSVM. The experimental results demonstrated that the MWV-SVM with GRB kernel achieves the best classification accuracy of 88.2%. The combination of color histogram, Unser's texture, and shape features are more effective than any single kind of feature in classification of fruits. Using PCA to reduce features, we tested three different multi-class SVMs (WTA-SVM, MWV-SVM, and DAG-SVM) with linear kernel, *d*th Homogeneous Polynomial kernel, and Gaussian Radial Basis kernel in the dataset of 1,653 fruit images. The best results were obtained for MWV-SVM with the GRB kernel with an overall classification accuracy of 88.2%. Future research will concentrate on: (1) extending our research to sliced, dried, canned, and tinned fruits; (2) include additional features to increase the classification accuracy; (3) accelerate the algorithm; and (4) find distinguishable features for Bosc Pears, Passion Fruits, and Avocado.

## Figures and Tables

**Figure 3.**
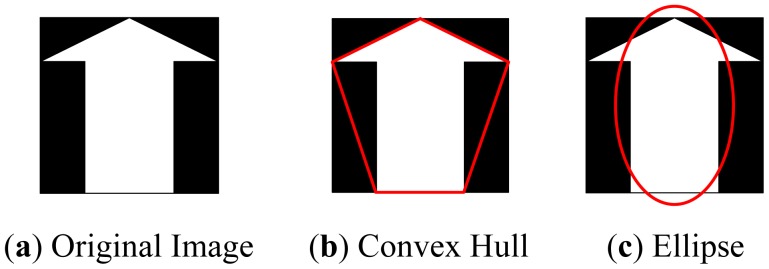
Illustration of the morphology measures.

**Table 1.** Sum and difference histogram based measures.

Measure	Formula
Mean (*μ*)	*μ* = (1/2)× Σ*_i_ ih_s_* (*i*; *δ*_1_, *δ*_2_)
Contrast (*C_n_*)	*C_n_* = Σ*_j_ j*^2^*h_d_* (*j*; *δ*_1_, *δ*_2_)
Homogeneity (*H_g_*)	*H_g_* = Σ*_j_* (1/(1+ *j*^2^))*h_d_* (*j*; *δ*_1_, *δ*_2_)
Energy (*E_n_*)	*E_n_* = Σ*_i_ h_s_* (*i*; *δ*_1_, *δ*_2_)^2^ Σ*_j_ h_d_* (*j*; *δ*_1_, *δ*_2_)^2^
Variance (*σ*^2^)	*σ*^2^ = (1/2)×(Σ*_i_*(*i* − 2*μ*)^2^) *h_s_* (*i*; *δ*_1_, *δ*_2_)+ Σ*_j_ j*^2^*h_d_* (*j*; *δ*_1_, *δ*_2_))
Correlation (*C_r_*)	*C_r_* = (1/2)×(Σ*_i_*(*i* − 2*μ*)^2^) *h_s_* (*i*; *δ*_1_, *δ*_2_)− Σ*_j_ j*^2^*h_d_* (*j*; *δ*_1_, *δ*_2_))
Entropy (*H_n_*)	*H_n_* = − Σ*_i_ h_s_* (*i*; *δ*_1_, *δ*_2_)log(*h_s_* (*i*; *δ*_1_, *δ*_2_))− Σ*_j_ h_d_*(*j*; *δ*_1_, *δ*_2_)log *h_d_*(*j*; *δ*_1_, *δ*_2_))

**Table 2.** The Morphology based Measures.

Measure	Meaning
Area (*A_r_*)	The actual number of pixels inside the object
Perimeter (*P_r_*)	The distance around the boundary of the object
Euler (*E_l_*)	The Euler number of the object
Convex (*C_n_*)	The number of pixels of the convex hull
Solidity (*S_l_*)	The proportion of area to convex hull
MinorLength (*M_n_*)	The length of the minor axis of the ellipse
MajorLength (*M_j_*)	The length of the major axis of the ellipse
Eccentricity (*E_c_*)	The eccentricity of the ellipse

**Table 3.** Four Common Kernels.

Name	Formula	Parameter(s)
Homogeneous Polynomial (HPOL)	*k*(*x_i_*, *x_j_*) = (*x_i_* · *x_j_*)*^d^*	***d***
Inhomogeneous Polynomial	*k*(*x_i_*, *x_j_*) = (*x_i_* · *x_j_*+1)*^d^*	***d***
Gaussian Radial Basis (GRB)	*k*(*x_i_*, *x_j_*) = exp (−*γ*‖*x_i_* − *x_j_*‖^2^)	*_γ_*
Hyperbolic Tangent	*k*(*x_i_*, *x_j_*) = tanh(*κx_i_* · *x_j_ + c*)	*_κ_*, ***c***

**Table 4.** Samples of *Fruit* dataset of 18 different categories.

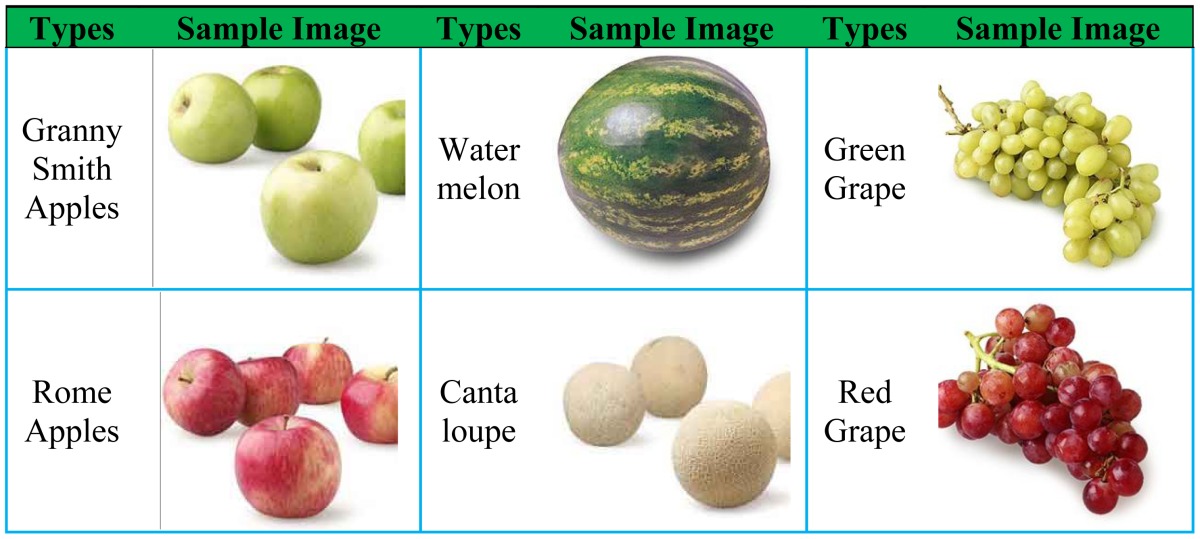 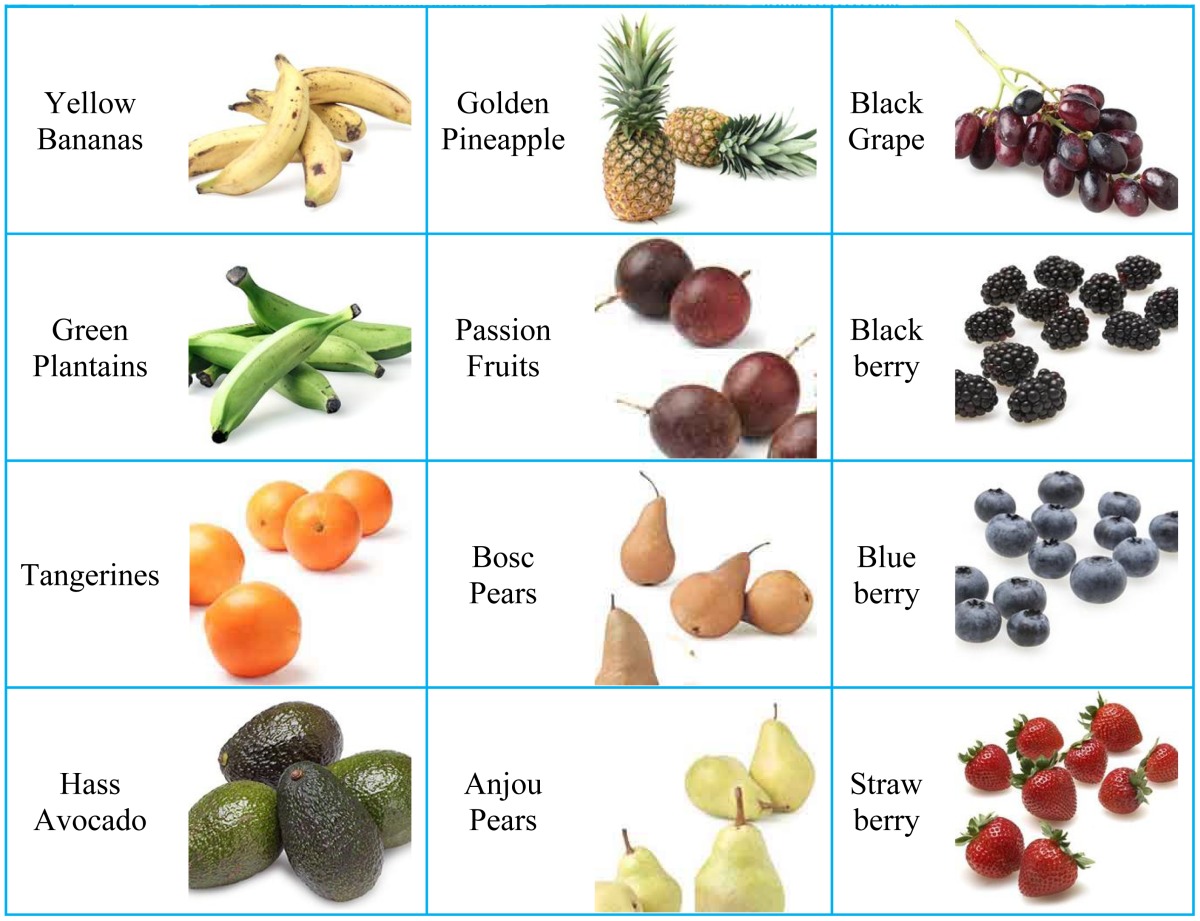

**Table 5.** The cumulative variances of PCA-transformed new features.

Dimensions	1	2	3	4	5	6	7	8	9	10
**Variance (%)**	**30.73**	**54.36**	**66.00**	**73.60**	**79.43**	**83.73**	**86.82**	**88.68**	**90.15**	**91.45**
**Dimensions**	**11**	**12**	**13**	**14**	**15**	**16**	**17**	**18**	**19**	**20**
**Variance (%)**	**92.55**	**93.51**	**94.35**	**95.08**	**95.71**	**96.27**	**96.76**	**97.19**	**97.55**	**97.87**

**Table 6.** Classification Accuracy of SVMs.

	LIN	HPOL	GRB
**WTA-SVM**	48.1%	61.7%	55.4%
**MWV-SVM**	53.5%	75.6%	**88.2%**
**DAG-SVM**	53.5%	70.1%	84.0%

**Table 7.** Computation Time of SVMs (s).

	LIN	HPOL	GRB
**WTA-SVM**	8.439	9.248	11.522
**MWV-SVM**	1.680	1.732	1.917
**DAG-SVM**	0.489	0.403	0.563
